# Subclinical impairment of dynamic left ventricular systolic and diastolic function in patients with obstructive sleep apnea and preserved left ventricular ejection fraction

**DOI:** 10.1186/s12890-020-1099-9

**Published:** 2020-03-29

**Authors:** Antonello D’Andrea, Angelo Canora, Simona Sperlongano, Domenico Galati, Serena Zanotta, Giorgio Emanuele Polistina, Carmine Nicoletta, Giacomo Ghinassi, Maurizio Galderisi, Alessandro Sanduzzi Zamparelli, Patrizio Lancellotti, Marialuisa Bocchino

**Affiliations:** 1 Unit of Cardiology and Intensive Care, Umberto I Hospital, Viale San Francesco, 84014 Nocera Inferiore (Salerno), Italy; 2 Department of Clinical Medicine and Surgery, Respiratory Medicine Section, Federico II University (at Monaldi Hospital), Via L. Bianchi, 5, 80131 Naples, Italy; 3 Unit of Cardiology, Department of Translational Medical Sciences, University of Campania “Luigi Vanvitelli”, Monaldi Hospital, Naples, Italy; 4 Haematology-Oncology and Stem Cell Transplantation Unit, Department of Haematology and Innovative Therapies, Istituto Nazionale Tumori- IRCCS Fondazione G. Pascale, Naples, Italy; 5 Department of Advanced Biomedical Sciences, Federico II University Hospital Via S. Pansini 5, Naples, Italy; 6 CHU de Liége, Service de Cardiologie, Liege, Belgium

**Keywords:** Obstructive sleep apnea, Exercise echocardiography, 2D speckle tracking echocardiography, Diastolic function, Oxidative burst

## Abstract

**Background:**

Hypoxia affects myocardial oxygen supply resulting in subclinical cardiac dysfunction in obstructive sleep apnea (OSA) patients, with cardiovascular complications being associated with increased oxidative burst (OB). The aims of our study were to assess left ventricular (LV) dynamic myocardial deformation and diastolic reserve at rest and upon exercise, along with OB determination in this patients subset.

**Methods:**

Conventional echocardiography, Doppler myocardial imaging and LV 2D speckle tracking echocardiography were performed in 55 OSA patients with preserved LV ejection fraction (EF) and 35 age and sex-comparable healthy controls. Peripheral OB levels were evaluated by flow cytometry.

**Results:**

Despite comparable LVEF, LV global longitudinal strain (GLS) was significantly reduced in OSA at rest (− 13.4 ± 3.8 vs − 18.4 ± 3.3 in controls, *P* <  0.001) and at peak exercise (− 15.8 ± 2.6 vs − 23.4 ± 4.3, *P* <  0.001). Systolic pulmonary artery pressure (sPAP) and E/E′ ratios increase during effort were higher in OSA than in controls (ΔsPAP 44.3% ± 6.4 vs 32.3% ± 5.5, *P* <  0.0001, and ΔE/E’ 87.5% ± 3.5 vs 25.4% ± 3.3, *P* <  0.0001, respectively). The best correlate of E/E′ at peak stress was peak exertion capacity (*r* = − 0.50, *P* <  0.001). OB was also increased in OSA patients (*P* = 0.001) but, unlike OSA severity, was not associated with LV diastolic dysfunction.

**Conclusions:**

Evaluation of diastolic function and myocardial deformation during exercise is feasible through stress echocardiography. OSA patients with preserved LVEF show subclinical LV systolic dysfunction, impaired LV systolic and diastolic reserve, reduced exercise tolerance, and increased peripheral levels of OB. Therapy aimed at increasing LV diastolic function reserve might improve the quality of life and exercise tolerability in OSA patients.

## Background

Obstructive sleep apnea (OSA) is characterized by repeated episodes of partial or complete upper airways collapse, resulting in apnea and hypopnea events, intermittent hypoxemia and frequent arousals [1]. OSA is an increasing health problem, often associated with cardiovascular disorders, including left (LV) and right ventricular (RV) dysfunction, arterial hypertension, coronary artery disease, heart failure, and arrhythmias [2, 3]. Repetitive hypoxia episodes adversely affect the interaction between myocardial oxygen demand and supply, with development of subclinical systolic dysfunction [4]. Furthermore, hormonal and metabolic dysregulation, oxidative burst (OB), systemic inflammation and mechanical hemodynamic disturbances lead to LV remodeling and diastolic dysfunction [5–8]. There is evidence of a consistent relationship between OB and OSA [9]. OB has been suggested as a marker of upper airway obstructive episodes and hypoxemia causing local oropharyngeal inflammation [10], and has been associated with cardiovascular complications [11, 12].

On this basis, we hypothesized that OSA patients with preserved LV ejection fraction (LVEF) would have dynamic abnormalities in LV myocardial deformation and/or increased dynamic diastolic stiffness. The aims of our study were to assess, by means of 2D speckle tracking echocardiography (2DSTE) and diastolic parameters, LV myocardial deformation and diastolic function indexes both at rest and during exercise, mainly focusing on the systolic and diastolic reserve. Reproducibility of 2DSTE measurements was also assessed. In addition, we evaluated peripheral levels of OB as we aimed to address any relationship with basal and dynamic heart function parameters.

## Methods

### Study population

From October 2017 to May 2018, 55 consecutive patients affected by moderate-severe OSA, referred to our Respiratory Medicine Division, were enrolled. Patients with a clinical history of concomitant lung disease (including chronic obstructive pulmonary disease, bronchial asthma, interstitial lung diseases), coronary artery disease, valvular heart disease, congestive heart failure, arrhythmias, and pulmonary hypertension were excluded. All OSA patients were not being treated with continuous positive airway pressure (CPAP). Thirty-five age- and sex-comparable healthy subjects referred to our attention for a voluntary cardiovascular screening were enrolled as controls. The study was conducted in accordance with the amended Declaration of Helsinki. The local Ethics committee approved the study and all individuals gave written informed consent.

### OSA assessment

OSA diagnosis was performed with home overnight cardiorespiratory polygraphy using a VitalNight data acquisition and analysis system (AirLiquide Medical System, Rangendigen, Germany) according to reference guidelines [13]. Data were analysed by trained sleep physicians with more than 3-yr experience and OSA severity was graded with the apnea/hypopnea index (AHI) according to accepted criteria [13]. Nocturnal respiratory failure (NRF) was defined if t90% was ≥30% or there was at least one period of 5 min minimum with a SpO_2_ ≤ 90% with a nadir of 85% during registration [14, 15]. t90 is the percentage of time spent with a SpO_2_ < 90%.

### Lung function

Spirometry, lung volumes measurement and determination of the haemoglobin (Hb)-adjusted single-breath diffusing lung capacity of the carbon monoxide (DLCO_sb_) were performed using a computer-assisted spirometer (Quark PFT 2008 Suite Version Cosmed Ltd., Rome, Italy) according to international standards [16–18]. The 6 min walking test (6MWT) was performed by trained hospital staff according to guidelines [19]. Arterial blood gas analysis at rest was also obtained.

### Heart study

Standard trans-thoracic echocardiography, Doppler evaluation and strain analysis were performed at rest and at peak effort using market available equipment (Vivid E9 - GE Healthcare, Milwaukee, WI, USA; MyLab Alpha ESAOTE, Florence, Italy). All measurements were performed at our Cardiology Unit and independently assessed by two cardiologists expert in echocardiography, according to current recommendations [20–22]. *Rest echocardiography:* LV ejection fraction (EF) and LV global longitudinal strain (GLS) were evaluated as systolic function indexes. LVEF was calculated using the Simpson biplane method, from the apical 4- and 2-chamber views. A biplane LVEF ≥52% for men and ≥ 54% for women were considered normal [20]. For strain calculation, the endocardial borders of the LV myocardial walls were traced by a point-and-click approach, in the end-systolic frame of the 2D images, from the apical 3-, 4-, and 2-chamber views. The tracking algorithm followed the endocardium during all the cardiac cycle. Basal, mid, and apical regions of interest were created and segments that failed to track were manually adjusted. Longitudinal strains for each of 18 segments were measured and LV GLS was calculated as the mean strain of all the segments. The tracking process and conversion to Lagrangian strains were performed offline using dedicated software (EchoPAC PC 2D strain, GE Healthcare, Milwaukee, WI, USA). We defined impaired GLS as > − 20% (a less negative value reflects a more impaired GLS) [20, 21]. The following diastolic function parameters were measured by pulsed wave (PW) Doppler and tissue Doppler imaging (TDI), in apical 4-chamber view: peak early (E) and late (A) diastolic velocity of the mitral inflow, E/A ratio, peak septal and lateral early myocardial diastolic velocity (e′) and average E/e′ ratio. The peak tricuspid regurgitation velocity (TRV) was measured in multiple echocardiographic windows. On the basis of the highest TRV obtained, systolic pulmonary artery pressure (sPAP) was calculated through the Bernoulli’s principle: (4xTRV^2^) + right atrial pressure (RAP). RAP was estimated by measuring the diameter of the inferior vena cava and its respiratory motion. Left atrial volume index (LAVI) was assessed through biplane area-length method, dividing the left atrial volume by the body surface area (BSA). Atrium acquisitions were made from the apical 4- and 2- chamber views. The presence of more than 2 between average E/e’ > 14, septal e’ < 7 cm/s or lateral e’ < 10 cm/s, TRV > 2.8 m/s (sPAP> 36 mmHg), and LAVI > 34 ml/m^2^, was considered expression of LV diastolic dysfunction in subjects with normal LVEF [22]. *Exercise stress echocardiography:* Standard supine bicycle exercise stress echocardiography was performed with incremental steps of 25 W every 2 min [23]. Parameters evaluated at peak exercise included LV GLS as systolic function index, LV diastolic parameters (E, septal and lateral e’ and average E/e’ ratio) and sPAP. An increase in the E/e’ ratio and/or sPAP upon exercise were considered expression of impaired LV diastolic function reserve [23].

### Oxidative burst determination

Oxidative burst of peripheral leukocytes was measured with the Phagoburst BURSTEST™ (PHAGOBURST™, BD Bioscences, La Jolla, CA, USA), according to the manufacturer’s instruction. Briefly, 100 μl of heparinized whole blood was incubated with opsonized *E.coli* at 37 °C for 10 min. A sample without stimulus served as negative background control. Dihydrorhodamine (DHR) 123 was added for 10 min to allow the conversion to fluorescent rhodamine 123 upon reactive oxygen species (ROS) production. After erythrocytes were removed and washing, 200 μl of DNA staining solution was added for 10 min to exclude aggregation artifacts. Samples were acquired with a FACS CANTO flow cytometer (BD Biosciences, La Jolla, CA, USA). Analysis was performed with the FACS DIVA software.

### Statistical analysis

Statistical analyses were performed using a commercially available package (SPSS, Rel. 21.0. 2016, SPSS Inc., Chicago, IL, USA). Variables are presented as mean ± standard deviation (SD). Two-tailed *t*-test for paired and unpaired data was used to assess changes between groups.

Linear regression analyses and partial correlation test by Pearson’s method were used to assess univariate relations. The following variables were included into the analysis: clinical parameters (age, heart rate, oxygen saturation, systolic blood pressure, diastolic blood pressure, body mass index (BMI), risk factors, co-morbidities); lung function, sleep-related and metabolic parameters; standard echocardiographic and 2DSTE parameters. To identify significant independent determinants of resting and dynamic LV diastolic function in OSA patients, their individual association with clinical, functional and echocardiographic variables was assessed by multivariate analysis, using a bidirectional stepwise regression. Variables included in our analysis were all the variables significantly (*p* <  0.05) associated with the explained variable by univariate analysis. Odds ratios (OR) were calculated using a logistic regression method, and Beta-coefficients were obtained by linear regression method. Values of *p* <  0.05 were considered significant for all analyses.

Receiver operating characteristic (ROC) curve analysis was performed to select optimal cut-off values of echocardiographic parameters. Reproducibility of GLS measurements was determined in all the subjects. Intra-observer variability and inter-observer variability were examined using the coefficient of variation (COV), defined as the ratio of the standard deviation (σ) to the mean (μ) (%), and by Bland-Altman analysis. COV, 95% confidence intervals (CIs), and percent errors were reported.

## Results

### Peripheral levels of oxidative burst are increased in OSA patients

**Fig. 1:**
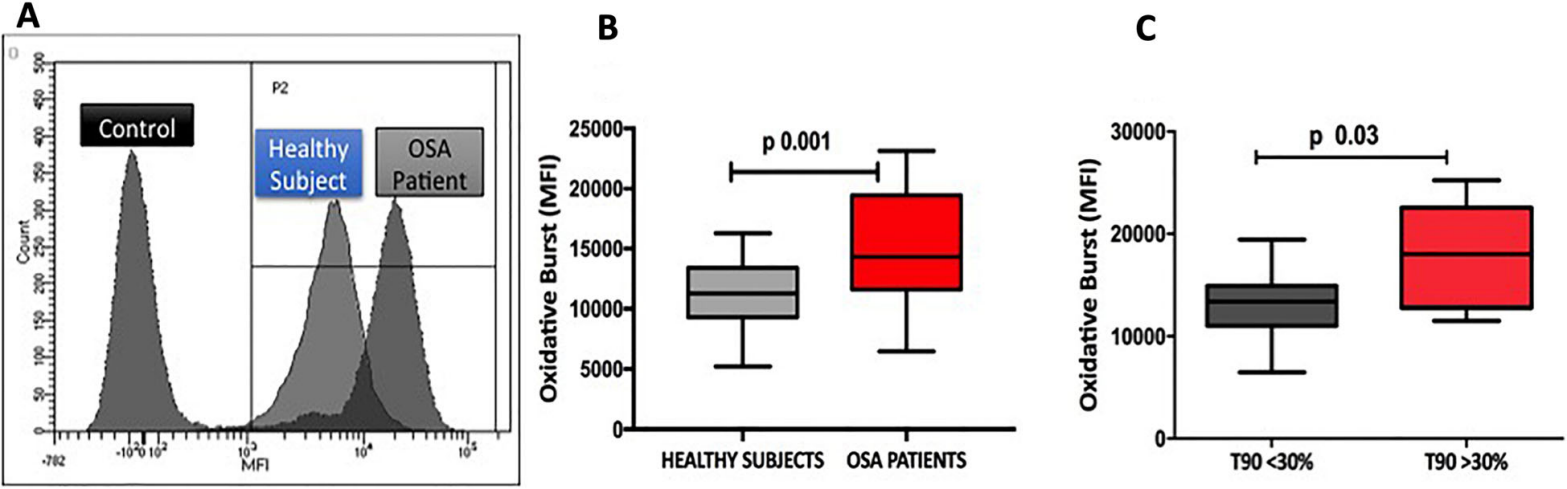
Levels of oxidative burst in OSA patients and healthy controls. **a** Distribution of oxidative burst (OB), calculated as mean fluorescence intensity (MFI), in OSA patients with respect to healthy volunteers; **b** Boxplot showing that peripheral levels of OB (MFI) are significantly increased in OSA patients as compared to healthy volunteers; **c** Boxplot showing the distribution of OB (MFI) in OSA patients according to t90%. As reported, OB levels are significantly increased in patients with t90 > 30%

Our study population was composed of 90 subjects, including 55 OSA patients and 35 healthy controls. Main demographic and clinical characteristics are summarized in Table 1. Lung function was preserved in both study groups (Table 1), with the exception of forced vital capacity that was slightly lower in OSA patients. Distribution of sleep-related respiratory events is reported in Table 1. Thirty patients were suffering from severe OSA (54%), while 21 (39%) had a concomitant condition of NRF, as assessed by a t90 > 30% in all cases. In line with previous observations [9–11], levels of OB were significantly increased in OSA patients than in controls (Fig. 1). OB was significantly higher in patients with a t90 > 30%, with no differences according to the AHI.

**Table 1:** Demographics and clinical features, lung function parameters, and sleep-related findings of the study population

Variable	OSA	Controls	*P*-value
	(*n* = 55)	(*n* = 35)	
Age (years)	54.9 ± 8.8	50.2 ± 5.4	NS
Gender, male sex	39 (71)	24 (68)	NS
Smoking habit			
Smokers	9 (16)	0 (0)	
Former smokers	23 (42)	0 (0)	
No smokers	23 (42)	35 (100)	
Pack/yr	32.8 ± 24.6		
BMI (Kg/m^2^)	32.9 ± 6.7	28.3 ± 4.7	**< 0.01**
Dyslipidemia (%)	10 (18)	0 (0)	
Hypertension (%)	14 (25)	0 (0)	
Diabetes (%)	12 (22)	0 (0)	
**Lung function parameters**			
FVC (%pred.)	85.4 ± 13.6	98 ± 12.5	**< 0.001**
FEV_1_ (%pred.)	87 ± 16.8	86 ± 13	NS
FEV_1_/FVC (%)	102.7 ± 15.4	84 ± 9.5	**< 0.001**
TLC (% pred)	85 ± 5.9	86 ± 3.8	NS
DLCO_sb_(% pred)	84.3 ± 16.1	86 ± 5	NS
pH	7.38 ± 0.3	7.39 ± 0.4	NS
PaO_2_ (mmHg)	84.7 ± 8.1	85.3 ± 6.2	NS
PaCO_2_ (mmHg)	39.8 ± 4.1	38.6 ± 4.5	NS
Lactates (mmol/L)	1.2 ± 1.4	1.1 ± 1.2	NS
HCO3^−^ (mmol/L)	26.1 ± 1.6	25.2 ± 1.4	NS
6MWT mt	512.1 ± 175.1	568 ± 145.6	NS
**Sleep-related events**			
TST (minutes)	358.4 ± 68.7	362 ± 64.5	NS
Supine time (%)	49.7 ± 33.8	41.2 ± 29.8	NS
AHI	46.3 ± 28.7	3.2 ± 1.2	< 0.0001
Supine AHI (events/hour)	41.4 ± 27	2.2 ± 2.4	< 0.0001
ODI	46 ± 30.7	3.3 ± 1.2	< 0.0001
t90 (%)	24 ± 22.2	0.4 ± 0.6	0.001
Nadir % SpO_2_	68.5 ± 11.5	83.2 ± 6.2	0.0001

Data are expressed as absolute number (%) or mean ± SD. Statistically significant results (*p* <  0.05) are reported in bold

Abbreviations: *BMI* Body mass index, *FVC* Forced vital capacity, *FEV*_*1*_ Forced expiratory volume in the 1st second, *TLC* Total lung capacity, *DLCO*_*sb*_ Single breath carbon monoxide lung diffusing capacity, *PaO*_*2*_ Oxygen arterial partial pressure, *PaCO*_*2*_ Carbon dioxide arterial partial pressure, *HCO3* Sodium bicarbonates, *6MWT mt*, Meters traveled during 6 min walking test, *TST* Total sleep time, *AHI* Apnea/hypopnea index, *ODI* Oxygen desaturation index, *t90%* Percentage of time spent with SpO_2_ < 90%, *SpO*_*2*_ Arterial oxygen saturation

### LV systolic function is impaired at rest and at peak exercise in moderate-severe OSA with a good reproducibility of 2DSTE measurements

**Fig. 2:**
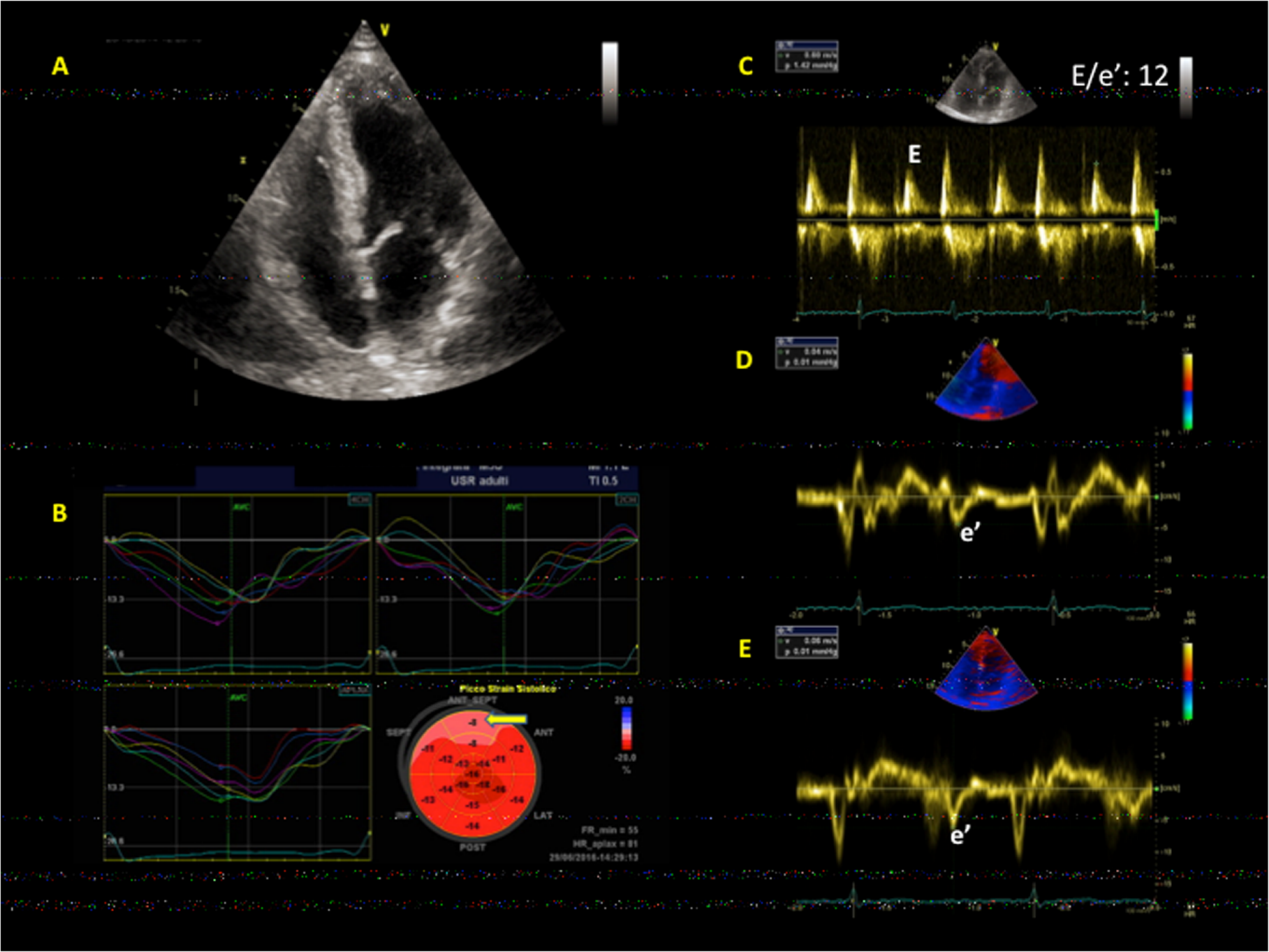
Left ventricular systolic and diastolic dysfunction in OSA patients. Two-dimensional echocardiography (**a**: apical four chamber view) showing mild impairment of resting LV regional and global strain (mainly in the septal region, see arrow) (**b**) and significant diastolic dysfunction assessed by transmitral flow pattern (**c**) and both lateral (**d**) and septal (**e**) pulsed Doppler tissue imaging

LVEF was similar in OSA and control cohorts, and preserved in both (Table 2). LV mass was mildly increased in OSA with no significant difference in LV diameters in the 2 groups (Table 2). LV speckle tracking was obtainable at rest in 95% of the total analysed segments. The remaining 5% segments were not considered due to a suboptimal tracking score. In OSA patients, LV GLS was significantly reduced at rest, this variation being statistically meaningful in all segments analysed (Table 2, Fig. 2). Also LV GLS increase upon exercise was lower as compared to controls (Table 3).

**Table 2:** Left ventricle standard echo and 2D speckle tracking echo measurements at rest and peak effort

Variable	OSA	Controls	*P*-value
	(*n* = 55)	(*n* = 35)	
**Resting measurements**			
SpO_2_ (%)	94.7 ± 16.2	98 ± 1.6	**< 0.01**
IVSd (mm)	11.4 ± 1.8	9.1 ± 2.3	**< 0.01**
PWd (mm)	10.4 ± 1.6	8.7 ± 2.1	**< 0.01**
LVEDD (mm)	48.3 ± 3.9	47.2 ± 4.4	NS
LVESD (mm)	34.3 ± 3.6	32.4 ± 4.1	NS
LV mass index (g/m^2^)	52.8 ± 5.3	48.1 ± 3.4	**< 0.01**
Biplane LVEF (%)	56.5 ± 6.2	57.4 ± 5.5	NS
LV GLS (%)	−13.4 ± 3.8	−18.4 ± 3.3	**< 0.001**
Mitral E velocity (cm/s)	0.9 ± 0.3	0.8 ± 0.4	NS
Mitral A velocity (cm/s)	0.7 ± 0.4	0.9 ± 0.3	NS
E/A ratio	1.2 ± 0.4	0.9 ± 0.4	**< 0.01**
Mitral septal e’ velocity (cm/s)	0.13 ± 0.02	0.16 ± 0.05	**< 0.01**
Mitrallateral e’ velocity (cm/s)	0.14 ± 0.03	0.17 ± 0.03	**< 0.01**
E/e’ ratio	8.2 ± 3.1	5.9 ± 2.8	**< 0.01**
LAVI (ml/m^2^)	32.4 ± 4.4	28.3 ± 5.1	**< 0.01**
sPAP (mmHg)	31.5 ± 7.8	21.3 ± 2.9	**< 0.01**
TAPSE (mm)	22.5 ± 3.3	24.5 ± 3.8	NS
Tricuspid S′ velocity (cm/s)	13.3 ± 2.2	14.4 ± 3.1	NS
**Peak effort measurements**			
SpO_2_ (%)	92.3 ± 3.2	97.2 ± 2.6	**< 0.01**
Exercise capacity (Watt)	115.3 ± 25	150.4 ± 35	**< 0.001**
Biplane LVEF (%)	62.3 ± 5.8	65.7 ± 6.8	NS
LV GLS (%)	−15.8 ± 2.6	−23.4 ± 4.3	**< 0.001**
Mitral E velocity (cm/s)	1.1 ± 0.5	1.2 ± 0.6	NS
Mitral A velocity (cm/s)	0.9 ± 0.4	0.9 ± 0.3	NS
E/A ratio	1.2 ± 0.4	1.3 ± 0.5	**< 0.01**
Mitral septal e’ velocity (cm/s)	0.08 ± 0.02	0.18 ± 0.05	**< 0.0001**
Mitral lateral e’ velocity (cm/s)	0.07 ± 0.03	0.19 ± 0.03	**< 0.0001**
E/e’ ratio	15.4 ± 4.1	7.4 ± 2.8	**< 0.0001**
sPAP (mmHg)	45.5 ± 5.8	28.4 ± 4.2	**< 0.001**
TAPSE (mm)	26.4 ± 2.3	27.5 ± 5.8	NS
Tricuspid s’ velocity (cm/s)	16.4 ± 3.1	17.7 ± 4.1	NS

Data are expressed as mean ± SD. Statistically significant results (*p* < 0.05) are reported in bold

Abbreviations*: SpO*_*2*_ Arterial oxygen saturation, *IVSd* Inter-ventricular septum thickness at end diastole, *PWd* Posterior wall thickness at end diastole, *LVEDD* Left ventricular end diastolic diameter, *LVESD* Left ventricular end systolic diameter, *LV* Left ventricle, *LVEF* Left ventricular ejection fraction, *LV GLS* Left ventricular global longitudinal strain, *LAVI* Left atrial volume index, *sPAP* Systolic pulmonary artery pressure, *TAPSE* Tricuspid annular plane systolic excursion

**Table 3:** Changes in echocardiographic parameters and oxygen saturation in OSA and controls during effort

Variable	OSA	Controls	*P*-value
	(*n* = 55)	(*n* = 35)	
Δ sPAP (%)	44.3 ± 6.4	32.3 ± 5.5	**< 0.001**
Δ E/e’ ratio (%)	87.5 ± 3.5	25.4 ± 3.3	**< 0.0001**
Δ LV GLS (%)	15.8 ± 3.4	25.4 ± 4.1	**< 0.001**
Δ SpO_2_ (%)	- 2.5 ± 3.3	−0.8 ± 2.8	**< 0.01**

Data are expressed as mean ± SD. Statistically significant results (*p* < 0.05) are reported in bold

Abbreviations*: sPAP* Systolic pulmonary artery pressure, *LV GLS* Left ventricular global longitudinal strain, *SpO*_*2*_ Arterial oxygen saturation

Overall, we observed a good reproducibility of global LV strain measurements in our study population, similar to that reported for speckle-tracking-based strain measures performed in selected samples – including healthy young adults [20, 21]. The average coefficients of variation were ≤ 7% for the longitudinal strain measures. For all analyzed measures, intra- and intra-observer ICC values were ≥ 0.80 **(**Intra-observer variability: COV: LV GLS: 5.37 (ICC 0.73); Bland-Altman analysis: LV GLS (95% CI ± 1.5; percent error 3.2%). Inter-observer variability: COV: LV GLS: 7.22 (ICC 0.77); Bland-Altman analysis: LV GLS (95% CI ±1.7; percent error 3.6%).

### LV diastolic function is impaired at rest and at peak exercise in moderate-severe OSA

E/e’ ratio, sPAP and LAVI at rest were all significantly higher in patients affected by OSA as compared to healthy controls; moreover, both septal and lateral e’ velocities were significantly lower in the former (Table 2, Fig. 2). According to recent guidelines, a definite diastolic dysfunction was present at rest in 19 OSA (34.5%) [22].

E/e’ increase during exertion was significantly higher in patients affected by OSA when compared to controls; sPAP increase (difference between peak exertion and resting sPAP) also showed the same trend along with a three-fold decrease of oxygen saturation (Table 3).

### Exercise tolerance is reduced in moderate-severe OSA

During physical exercise, OSA patients showed a reduced tolerance with a lower maximal workload in Watts (115.3 ± 25 vs 150.4 ± 35, *p* <  0.001) and oxygen saturation level (92.3% ± 3.2 vs 97.2% ± 2.6, *p* <  0.01) as compared to healthy controls (Table 2).

### Resting and exercise-induced LV diastolic dysfunction is associated with OSA severity but not with OB levels

Logistic regression analysis showed that arterial hypertension (OR 2.67, 95% CI 1.74–5.61; *p* <  0.01), resting LV GLS > − 15% (OR 3.3, 95% CI 2.34–4.15; *p* <  0.001) and severe AHI (OR 2.96, 95% CI 1.92–4.91; *p* <  0.001) were independently associated with LV diastolic dysfunction at rest.

At peak exercise, by linear regression analysis E/e’ ratio was directly related to peak exertion capacity expressed in Watts (beta coefficient − 0.50, *p* <  0.001), to blood lactates at rest (beta coefficient 0.36, *p* <  0.01), to resting LV GLS (beta coefficient 0.46, *p* <  0.001), and to the AHI (beta coefficient 0.42, *p* <  0.001) (Table 4). No significant correlations were found with OB, BMI smoking, and co-morbidities.

**Table 4:** Multivariate analysis model: correlation between E/e’ ratio at peak exercise with univariable clinical parameters

Variable		Beta coefficient	*P*-value
**LV E/e’ at peak exercise**	**Watts (at peak effort)**	− 0.50	**< 0.001**
	**Blood lactates (at rest)**	0.36	**< 0.01**
	**LV GLS (at rest)**	0.46	**< 0.001**
	**AHI**	0.42	**< 0.001**

Statistically significant results (*p* < 0.05) are reported in bold

Abbreviations: *LV GLS* Left ventricular global longitudinal strain, *AHI* Apnea-hypopnea index

## Discussion

OSA is frequently associated with adverse clinical outcomes [1], including cardiovascular diseases [24–27]. In the present study, we showed that OSA patients: (1) have subclinical LV systolic dysfunction despite a preserved LV output, and a low contractile reserve as compared to healthy controls; (2) have an impaired LV diastolic reserve along with a reduced exercise tolerance; (3) display higher levels of OB that are not correlated with diastolic heart function.

In 2007, Gondi et al. reported that OSA was associated with sleep-induced LV systolic longitudinal dysfunction, as measured by pulsed wave Doppler and tissue Doppler imaging (TDI) [28]. As TDI is technically limited by the insonation angle of the ultrasound beam, we further proved by 2DSTE, which is independent of the interrogation angle, that LV and RV early myocardial dysfunction occur in OSA patients and are associated with disease severity [29]. The relationship between OSA and LV systolic dysfunction is not surprising as OSA patients experience repeated increases in LV after-load during sleep, due to exaggerated negative intra-thoracic pressure and intermittent hypoxia and arousals. Acute repeated increases in after-load can therefore result in LV subclinical systolic dysfunction [29]. In this study, we enrolled patients with moderate-severe OSA and healthy controls with preserved LVEF and found an early involvement of the LV myocardium in the former. Actually, despite LVEF, that is traditionally used as systolic function parameter, was normal in OSA patients, we found an impaired basal LV GLS, which is expression of subclinical LV pump dysfunction. In addition, we also found that strain increase during physical exercise in OSA patients was lower if compared to healthy controls, reflecting their low contractile reserve. Interestingly, we previously detected that OSA patients have also a subclinical RV dysfunction, and that RV GLS impairment was associated with sPAP and disease severity [30].

Previous studies show that the prevalence of LV diastolic dysfunction at rest among OSA patients varies from 23 to 56%, depending on the sample size and the method of diastolic function assessment [31–34]. In our cohort we observed that, according to recent guidelines [22], a definite diastolic dysfunction was present in the 34.5% of OSA patients at rest. Pathophysiological mechanisms underlying LV diastolic dysfunction in OSA have been widely studied: repetitive hypoxemia/re-oxygenation sequences, sympathetic bursts, renin-angiotensin-aldosterone system activation, oxidative stress, systemic inflammation, intra-thoracic pressure reduction and trans-mural pressure increasing, all participate in the development of LV hypertrophy and remodelling, and hence of LV diastolic dysfunction [5–8].

To our knowledge this is the first study addressing the LV diastolic performance both at rest and at peak exercise in a selected population of OSA patients. In middle-aged healthy subjects, the E/e′ ratio does not change significantly with exercise because of proportional increases in both the mitral inflow and annular velocities [35–38]. Conversely, in our series almost all patients enrolled showed an increase of the E/e’ ratio, that was significantly higher than in the control group. This finding indicates an impairment of the LV diastolic function reserve resulting in an increased LV filling pressure during exercise [22, 39, 40]. In agreement with these observations, also sPAP increase was significantly higher in OSA than in healthy subjects. The mean sPAP value in OSA patients was > 43 mmHg (as estimated by a TRV > 3.1 m/s), which is the echocardiography threshold of abnormal diastolic stress according to international guidelines [23]. The present study clearly underlines the strong relation between E/e’ ratio and exercise tolerance in OSA. Patients with a higher E/e’ ratio at peak exercise, and hence a more impaired diastolic reserve, showed a reduced functional capacity highlighted by a lower maximal workload and a reduced peak SpO_2_ level. The E/e’ ratio at peak exercise also resulted significantly correlated to LV GLS, to AHI, which reflects OSA severity, and to blood lactates, which are suggestive of inadequate tissue perfusion. These findings can be explained by the mentioned pathological mechanisms responsible for myocardial remodelling and fibrosis. The latter provokes impairment in both systolic and diastolic function, which can worsen during exercise, leading to reduced functional capacity.

Our study has some limitations. First, it is a single-centre study with a sample size too small to drive definitive general conclusions concerning subclinical LV functional impairment in OSA patients. Also, our findings cannot necessarily be extrapolated to patients with mild disease. Finally, despite we found no correlations between the diastolic impairment during exercise and patients age, OB, BMI, smoking and co-morbidities, we cannot definitely exclude their contribution. Indeed, while we excluded OSA patients with major heart and lung diseases, the prevalence of cardiovascular risk factors, such as smoke, obesity, dyslipidemia, diabetes, and hypertension, was still not negligible in our patient cohort. Further studies in larger populations will certainly help to address their impact by ad hoc sub-group analyses.

## Conclusions

In conclusion, evaluation of diastolic function and myocardial deformation during exercise is feasible through stress echocardiography and provides valuable information in predicting exercise capacity in a selected population of moderate-severe OSA patients with preserved LVEF. These patients show subclinical LV systolic dysfunction, impaired LV systolic and diastolic reserve, reduced exercise tolerance, and increased peripheral levels of OB. A CPAP treatment and a therapy aimed at increasing LV diastolic function reserve might improve the quality of life and exercise tolerability in this target population.

## Data Availability

The datasets used and/or analysed during the current study are available from the corresponding author on reasonable request.

## Abbreviations

2DSTE: 2D speckle tracking echocardiography
6-MWT: 6-min walk test
A: Late diastolic velocity of the mitral inflow
AHI: Apnea/hypopnea index
BSA: Body surface area
CPAP: Continuous positive airway pressure (CPAP)
DHR: Dihydrorhodamine
DLCO_sb_: Single-breath diffusing lung capacity of the carbon monoxide
E: Early diastolic velocity of the mitral inflow
e’: Early myocardial diastolic velocity
EF: Ejection fraction
GLS: Global longitudinal strain
LAVI: Left atrial volume index
LV: Left ventricular
NRF: Nocturnal respiratory failure
OB: Oxidative burst
OSA: Obstructive sleep apnea
PW: Pulsed wave
RAP: Right atrial pressure
ROC: Receiver operating characteristic
ROS: Reactive oxygen species
RV: Right ventricular
sPAP: Systolic pulmonary artery pressure
SpO_2_: Oxygen saturation
TDI: Tissue Doppler imaging
TRV: Tricuspid regurgitation velocity
